# Modelling the Hindered Settling Velocity of a Falling Particle in a Particle-Fluid Mixture by the Tsallis Entropy Theory

**DOI:** 10.3390/e21010055

**Published:** 2019-01-11

**Authors:** Zhongfan Zhu, Hongrui Wang, Dingzhi Peng, Jie Dou

**Affiliations:** 1 Beijing Key Laboratory of Urban Hydrological Cycle and Sponge City Technology, College of Water Sciences, Beijing Normal University, Xinjiekouwai Street 19, Beijing 100875, China; 2 Public Works Research Institute, Minamihara 1-6, Tsukuba, Ibaraki-ken 305-8516, Japan

**Keywords:** entropy, Tsallis entropy, probability distribution, hindered settling velocity, particle-fluid mixture

## Abstract

The settling velocity of a sediment particle is an important parameter needed for modelling the vertical flux in rivers, estuaries, deltas and the marine environment. It has been observed that a particle settles more slowly in the presence of other particles in the fluid than in a clear fluid, and this phenomenon has been termed ‘hindered settling’. The Richardson and Zaki equation has been a widely used expression for relating the hindered settling velocity of a particle with that in a clear fluid in terms of a concentration function and the power of the concentration function, and the power index is known as the exponent of reduction of the settling velocity. This study attempts to formulate the model for the exponent of reduction of the settling velocity by using the probability method based on the Tsallis entropy theory. The derived expression is a function of the volumetric concentration of the suspended particle, the relative mass density of the particle and the particle’s Reynolds number. This model is tested against experimental data collected from the literature and against five existing deterministic models, and this model shows good agreement with the experimental data and gives better prediction accuracy than the other deterministic models. The derived Tsallis entropy-based model is also compared with the existing Shannon entropy-based model for experimental data, and the Tsallis entropy-based model is comparable to the Shannon entropy-based model for predicting the hindered settling velocity of a falling particle in a particle-fluid mixture. This study shows the potential of using the Tsallis entropy together with the principle of maximum entropy to predict the hindered settling velocity of a falling particle in a particle-fluid mixture.

## 1. Introduction

In sediment transport dynamics, the settling velocity of the sediment particle is an important parameter needed for modelling vertical sediment flux in rivers, estuaries, deltas and marine environments [1,2,3]. In a still and clear fluid, a falling particle can accelerate downward due to gravitational force and reach its terminal velocity when the upward drag and submerged weight are in balance [4,5]. Many models for predicting the settling velocity of the particle (not limited to sediment particles) have been proposed [1,2,4,5,6,7]. By contrast, for a sediment-laden flow, it has been observed that the settling velocity of a falling particle is reduced compared to the settling of a particle in still fluid due to increased suspension concentration [8,9,10], which has been termed ‘hindered settling’. The Richardson and Zaki [11] equation is a widely used expression for relating the hindered settling and the settling in clear fluid, as follows:

(1) $$\omega_{m}=\omega {\left(1-c\right)}^{n_{H}},$$

where $\omega_{m}$ and ω are the settling velocities of the sediment particle in a sediment-laden flow and in a clear fluid, respectively, c is the volumetric concentration of the sediment particle, and $n_{H}$ is the exponent of reduction of the settling velocity in a sediment-fluid mixture, which depends on the particle’s Reynolds number R given by $\omega d_{p}/\nu_{0}$, where $d_{p}$ is the particle’s diameter and $\nu_{0}$ is the kinematic viscosity of a clear fluid.

However, several experiments under various conditions have shown that the experimental settling velocity of the particle is lower than that predicted by Equation (1) [8,12,13]. To that end, many empirical or semi-empirical studies have been carried out to modify the expression of $n_{H}$ to a better accuracy with the experimental data. Garside and Al-Dibouni [14] proposed an empirical expression of $n_{H}$as$\left(5.1-n_{H}\right)/\left(n_{H}-2.7\right)=0.1R^{0.9}$, which shows good agreement with the experimental data. Chien and Wan [15] also showed that the exponent $n_{H}$ is a function of R. Cheng [12] pointed out that the exponent $n_{H}$ not only depends on the particle’s Reynolds number but also on the particle’s mass density and volumetric concentration. A theoretical form of the exponent $n_{H}$ as a function of the mass density of the particle and the volumetric concentration was derived by Pal and Ghoshal [8] by introducing the concept of the apparent diameter of a particle, and the derived expression has shown good agreement with previously published experimental data. Furthermore, Baldock et al. [16] carried out an experiment to investigate the settling velocity of sediment particles with different combinations of particle diameters and particle mass densities, whereas the study of Tomkins et al. [17] incorporated the effect of the shape of the sand grains. The segregating effect between the sediment grains and the mud flows was discussed in the numerical simulation work of Van and Bang [18].

These studies have provided some physical insights to the hindered settling process of the particle in a particle-fluid mixture. During recent decades, some researchers have adopted the probability method based on entropy theory to investigate some classic hydraulic engineering problems, including predicting the one-dimensional and two-dimensional velocity distributions in open channels [19,20,21], estimating the sediment concentration distribution [22,23,24,25,26] and calculating the shear stress [27,28]. Recently, Singh et al. [29] reviewed the progress in the application of the entropy theory into water engineering problems. These works indicated that the probability method-based on the entropy theory could provide an easy and feasible tool for predicting some hydraulic problems besides the traditional deterministic methods.

Recently, Kumbhakar et al. [13] have used the Shannon entropy theory to derive the expression of the exponent of reduction of the settling velocity of the particle in a particle-fluid mixture. Another entropy, called the Tsallis entropy, which is a generalization of the Shannon entropy, has not been adopted for estimating the exponent of reduction of settling velocity. Thus, it may be interesting to explore the hindered settling velocity based on the Tsallis entropy. This study attempts to derive an entropy-based expression for the exponent of reduction of the settling velocity of a particle in particle-fluid mixture, by using the Tsallis entropy theory. Section 2 derives the expression for the exponent of reduction of the settling velocity of a particle using the Tsallis entropy theory. The derived expression is tested against some experimental data collected from the literature in Section 3, and Section 4 contains the comparisons of the expression with some existing deterministic models and the Shannon entropy-based expression. Finally, Section 5 presents concluding remarks.

## 2. Methodology for Determination of the Exponent of Reduction of Settling Velocity

It is assumed that the exponent of reduction of settling velocity $n_{H}$ is a continuous random variable. Determination of the exponent of reduction of settling velocity using the Tsallis entropy entails the following steps: (1) definition of the Tsallis entropy; (2) specification of the constraints; (3) maximization of the entropy; (4) determination of the Lagrange multiplier; (5) the hypothesis regarding the cumulative probability distribution; and (6) derivation of the expression for the exponent of reduction of the settling velocity.

### 2.1. Definition of the Tsallis Entropy

Let the exponent of reduction of the settling velocity $n_{H}$ be the random variable with probability density function$f\left(n_{H}\right)$. The Tsallis function, $n_{H}$,$H\left(n_{H}\right)$, can be written as [30]

(2) $$H\left(n_{H}\right)=\frac{1}{m-1}\left\{1-\int_{n0}^{n_{1}}{\left[f\left(n_{H}\right)\right]}^{m}dn_{H}\right\}$$

where $n_{0}$ and $n_{1}$ are the lower and upper limits of $n_{H}$, respectively, and m is the entropy index and is a real number not equal to 1. The Tsallis entropy is a non-extensive entropy that can reduce to the Shannon entropy if the exponent m→1 in Equation (2) [31]. Theoretically, the Tsallis entropy is maximum when the probability density function is uniform within its limits. Equation (2) expresses a measure of uncertainty of $f\left(n_{H}\right)$ or the average information content of sample $n_{H}$.

### 2.2. Specification of Constraint

If the observations of $n_{H}$ are available, then we can express the information about the random variable in terms of constraints [31]. First, the total probability law must be satisfied for the probability density function $f\left(n_{H}\right)$, that is

(3) $$\int_{n0}^{n_{1}}f\left(n_{H}\right)dn_{H}=1.$$

The other constraint equation is given as

(4) $$\int_{n0}^{n_{1}}n_{H}f\left(n_{H}\right)dn_{H}=\bar{n_{H}},$$

where $\bar{n_{H}}$ is the mean value of $n_{H}$. Equation (4) is the mean constraint.

### 2.3. Maximization of Entropy 

It should be noted that there are an infinite number of probability density distributions satisfying the constraint equations, Equations (3) and (4). Thus, in order to choose among all of the distribution functions satisfying the constraint equations, we use the principle of the maximum entropy developed by Jaynes [32,33,34] in this study. This principle requires that the derived probability distribution is the one that corresponds to the maximum entropy or uncertainty. To that end, the method of the Euler-Lagrange calculus of variation was adopted [29]. Consequently, the Lagrangian function L can be written as follows:

(5) $$\begin{array}{l} L=\frac{1}{m-1}\left\{1-\int_{n0}^{n_{1}}{\left[f\left(n_{H}\right)\right]}^{m}dn_{H}\right\}-\lambda_{0}\left[\int_{n0}^{n_{1}}f\left(n_{H}\right)dn_{H}-1\right]\\ -\lambda_{1}\left[\int_{n0}^{n_{1}}n_{H}f\left(n_{H}\right)dn_{H}-\bar{n_{H}}\right] \end{array}$$

where $\lambda_{0}$ and $\lambda_{1}$ are two Lagrange multipliers that need to be determined from the constraint equations, Equations (3) and (4). Treating $n_{H}$as the independent variable and $f\left(n_{H}\right)$ as the dependent variable, the Euler-Lagrange equation becomes

(6) $$\frac{\partial L}{\partial f}-\frac{d}{dn_{H}}\left(\frac{\partial L}{\partial f^{\prime }}\right)=0,$$

where $f^{\prime }$ denotes the derivative of f with respect to $n_{H}$. From Equation (5) it is obvious that the Lagrangian function is not a function of $f^{\prime }$, thus Equation (6) becomes

(7) $$\frac{\partial L}{\partial f}=0\Rightarrow \frac{1}{m-1}\left[1-mf{\left(n_{H}\right)}^{m-1}\right]-\lambda_{0}-\lambda_{1}n_{H}=0,$$

leading to the following expression for $f\left(n_{H}\right)$:

(8) $$f\left(n_{H}\right)={\left[\frac{m-1}{m}\left(\frac{1}{m-1}-\lambda_{0}-\lambda_{1}n_{H}\right)\right]}^{\frac{1}{m-1}}$$

**Figure 1:**
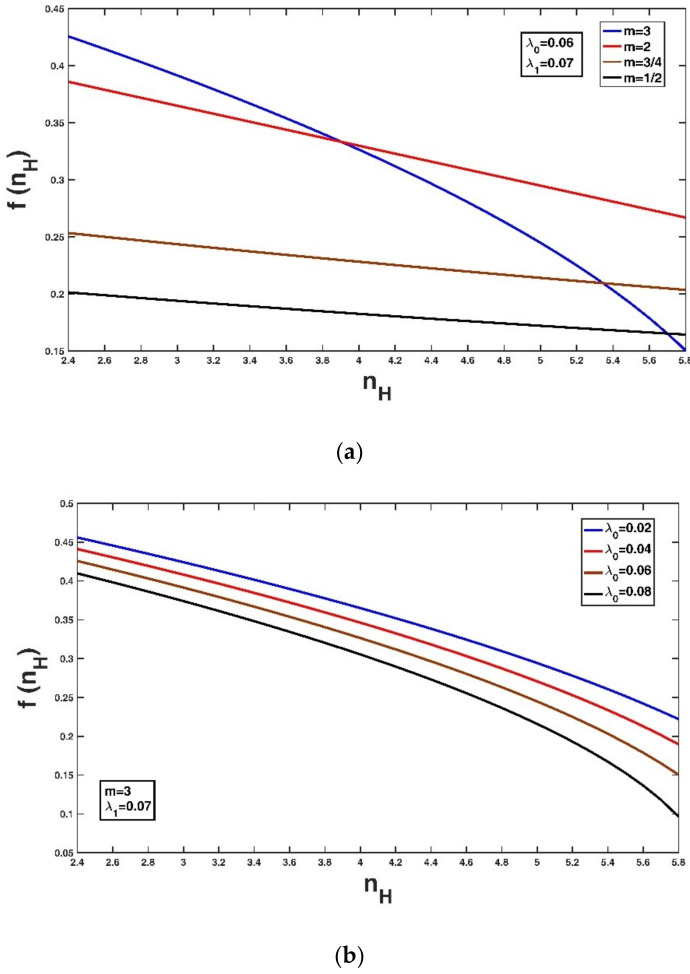

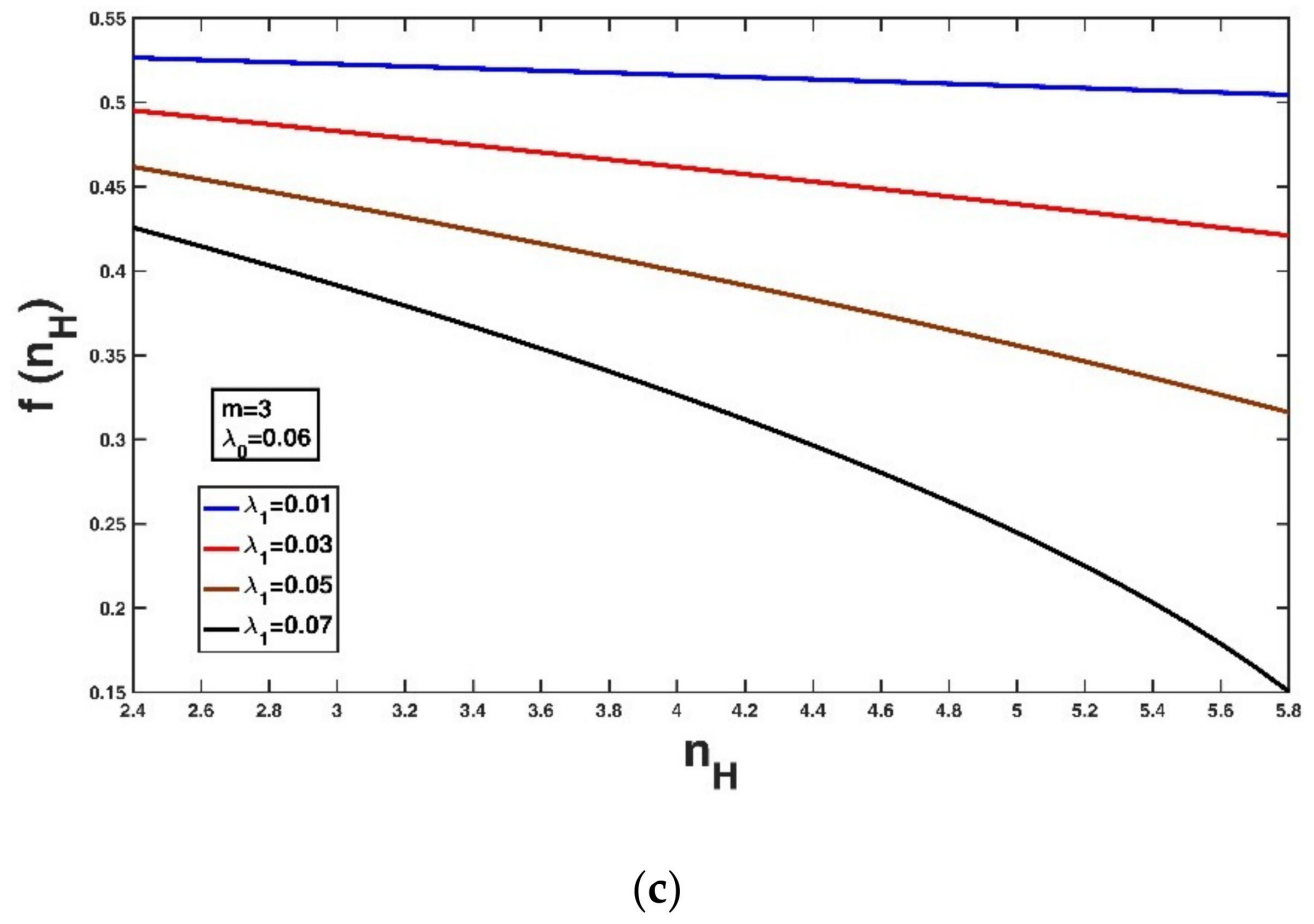
Variation of the probability density functions with different m (**a**), $\lambda_{0}$ (**b**) and $\lambda_{1}$ (**c**) when keeping other the values constant.

Assuming some fixed values of Lagrange multipliers, the variation of this probability density function $f\left(n_{H}\right)$ with m is shown in Figure 1a. It can be seen that when m is smaller than 2, $f\left(n_{H}\right)$ decreases almost linearly with the increase in $n_{H}$. Keeping one value fixed, the variation of the probability density function $f\left(n_{H}\right)$ with the other Lagrange multipliers is shown in Figure 1b,c. Figure 1b shows the variation of $f\left(n_{H}\right)$ with $\lambda_{0}$: as $\lambda_{0}$ decreases, $f\left(n_{H}\right)$ tends to increase at a fixed $n_{H}$ value. Figure 1c shows the variation of $f\left(n_{H}\right)$ with $\lambda_{1}$: as $\lambda_{1}$ increases, $f\left(n_{H}\right)$ tends to decrease at a fixed $n_{H}$.

Thus, the cumulative distribution function (CDF) of $n_{H}$,$F\left(n_{H}\right)$, can be obtained by simply integrating Equation (8) from $n_{0}$ to $n_{H}$ as

(9) $$F\left(n_{H}\right)={\left(\frac{m-1}{m}\right)}^{\frac{m}{m-1}}\frac{1}{\lambda_{1}}\left[{\left(\frac{1}{m-1}-\lambda_{0}-\lambda_{1}n_{0}\right)}^{\frac{m}{m-1}}-{\left(\frac{1}{m-1}-\lambda_{0}-\lambda_{1}n_{H}\right)}^{\frac{m}{m-1}}\right]$$

The maximum entropy function $H\left(n_{H}\right)$ is obtained by inserting Equation (8) into Equation (2) as follows:

(10) $$H\left(n_{H}\right)=\frac{1}{m-1}\left\{\begin{array}{l} \left(n_{1}-n_{0}\right)+{\left(\frac{m-1}{m}\right)}^{\frac{m}{m-1}}\frac{1}{\left(2m-1\right)}\frac{1}{\lambda_{1}}\\ \times \left[{\left(\frac{1}{m-1}-\lambda_{0}-\lambda_{1}n_{1}\right)}^{\frac{2m-1}{m-1}}-{\left(\frac{1}{m-1}-\lambda_{0}-\lambda_{1}n_{0}\right)}^{\frac{2m-1}{m-1}}\right] \end{array}\right\}$$

It can be seen from Equations (8)–(10) that all of the probability density functions, the CDF and the entropy function depend on two Lagrange multipliers,$\lambda_{0}$ and $\lambda_{1}$, which can be determined by the constraint equations.

### 2.4. Estimation of Lagrange Multipliers

Substituting Equation (8) into Equation (3) can yield

(11) $$\frac{1}{\lambda_{1}}{\left(\frac{m-1}{m}\right)}^{\frac{m}{m-1}}\left[{\left(\frac{1}{m-1}-\lambda_{0}-\lambda_{1}n_{1}\right)}^{\frac{m}{m-1}}-{\left(\frac{1}{m-1}-\lambda_{0}-\lambda_{1}n_{0}\right)}^{\frac{m}{m-1}}\right]=1$$

whereas by substituting Equation (8) into Equation (4), we obtain

(12) $$\begin{array}{l} n_{1}{\left(\frac{1}{m-1}-\lambda_{0}-\lambda_{1}n_{1}\right)}^{\frac{m}{m-1}}-n_{0}{\left(\frac{1}{m-1}-\lambda_{0}-\lambda_{1}n_{0}\right)}^{\frac{m}{m-1}}\\ +\frac{\left(m-1\right)}{\left(2m-1\right)}\frac{1}{\lambda_{1}}\left[{\left(\frac{1}{m-1}-\lambda_{0}-\lambda_{1}n_{1}\right)}^{\frac{2m-1}{m-1}}-{\left(\frac{1}{m-1}-\lambda_{0}-\lambda_{1}n_{0}\right)}^{\frac{2m-1}{m-1}}\right]\\ +\lambda_{1}\bar{n_{H}}{\left(\frac{m}{m-1}\right)}^{\frac{m}{m-1}}=0 \end{array}$$

It can be seen that Equations (11) and (12) constitute a non-linear equation system for the Lagrange multipliers $\lambda_{0}$ and $\lambda_{1}$. This equation system can be solved numerically for given values of $\bar{n_{H}}$,$n_{0}$,$n_{1}$ and the entropy index m by virtue of a non-linear equation solver in MATLAB software.

### 2.5. Hypothesis on the Cumulative Distribution Function 

To derive the explicit expression for the exponent of reduction of settling velocity in the real (space) domain, an equation connecting the probability domain to the space domain is needed [29]. Thus, a hypothesis regarding the CDF of the exponent of reduction of the settling velocity should be made so that the hypothesised CDF can reflect the characteristics of $n_{H}$. Several previous studies have shown that $n_{H}$ should be connected to the particle’s Reynolds number R, the volumetric concentration of the sediment and the mass density of the sediment [8,12]. In addition to this, the hypothesised CDF should have the following characteristics: (1) it is continuous and differentiable; (2) it is between 0 and 1; and (3) as R becomes large, CDF should approach zero, whereas as R decreases, CDF should approach 1. Although there are many types of CDFs satisfying the aforementioned characteristics, Kumbhakar et al. [13] proposed the following power-type hypothesis regarding the CDF to be an obvious and appropriate choice:

(13) $$F\left(n_{H}\right)=\exp\left[-A{\left(\frac{R}{c\Delta_{p}}\right)}^{\eta }\right],$$

where A and η are two parameters that should be positive real numbers, and $\Delta_{p}=s-1$ (here s is the ratio of the mass density of the sediment particles and that of the fluid).

### 2.6. Derivation of the Expression of $n_{H}$

By equating Equations (9) and (13) and using Equation (11), we can obtain the expression for the exponent of reduction of the settling velocity $n_{H}$ as

$$\begin{array}{l} n_{H}=-\frac{1}{\lambda_{1}}{\left\{\begin{array}{l} {\left(\frac{1}{m-1}-\lambda_{0}-\lambda_{1}n_{0}\right)}^{\frac{m}{m-1}}-\\ \left[{\left(\frac{1}{m-1}-\lambda_{0}-\lambda_{1}n_{0}\right)}^{\frac{m}{m-1}}-{\left(\frac{1}{m-1}-\lambda_{0}-\lambda_{1}n_{1}\right)}^{\frac{m}{m-1}}\right]\exp\left[-A{\left(\frac{R}{c\Delta_{p}}\right)}^{\eta }\right] \end{array}\right\}}^{\frac{m-1}{m}}\\ -\frac{\lambda_{0}}{\lambda_{1}}+\frac{1}{\lambda_{1}\left(m-1\right)} \end{array}$$

Equation (14) denotes the Tsallis entropy-based expression for the exponent of reduction of the settling velocity. The hindered settling velocity of a particle in the particle-fluid mixture can be estimated from Equations (1) and (14) for given values of R, c and $\Delta_{p}$.

## 3. Comparison with Existing Experimental Data

Eleven experimental data sets regarding the reduction of the settling velocity of the falling particle were collected from the literature to test the validity of the Tsallis entropy-based expression (Equation (14)), including beach sand (Wilhelm and Kwauk [35]), crushed sand (Fouda and Capes [36]), beach sand (Baldock et al. [16]), gravel (Baldock et al. [16]), filter sand (Cleasby and Woods [37]), glass spheres (Baldock et al. [16]), filter sand (Cleasby and Fan [38]), crushed flint (Cleasby and Fan [38]), filter sand (Baldock et al. [16]), crushed rock (Wilhelm and Kwauk [35]) and crushed sand (Jottrand [39]). These collected data are derived from different experiments by various researchers; they contain sand, gravel, glass, flint and crushed rock particles, varying in size $d_{p}$ from 0.06 to 3 mm and relative mass density of the particle s from 2.50 to 2.65. The data were chosen to cover both low and high particle Reynolds number conditions, R ranging from 0.22 to 1200.

By analysing these observation data, the lower limit, the upper limit and the mean value of the reduction of the settling velocity of the falling particle, $n_{0}$, $n_{1}$, $\bar{n_{H}}$ can be taken to be 2.40, 5.80 and 3.64, respectively. With these values taken from the collected experimental data, we can solve the non-linear equation system (Equations (11) and (12)) for the Lagrange multipliers $\lambda_{0}$ and $\lambda_{1}$ as $\lambda_{0}$ = 0.06 and $\lambda_{1}$ = 0.07 for m = 3. The value of m = 3 was simply adopted in this study, as adopted by some researchers [21,24,26]. Substituting these values into Equation (14) yields

(15) $$n_{H}=-14.29\times {\left\{0.15-0.14\times \exp\left[-A{\left(\frac{R}{c\Delta_{p}}\right)}^{\eta }\right]\right\}}^{\frac{2}{3}}+6.29.$$

To test the performance of the model developed against the collected experimental data and other models, an error analysis was carried out in this study by calculating the correlation coefficient k, defined as $k={\left[\mathrm{cov}\left(m,o\right)/\sigma_{m}\sigma_{o}\right]}^{2}$, and the normalised root mean square error (*NRMSE*), defined as $NRMSE=\sqrt{\frac{1}{N}\sum_{i=1}^{N}{\left(m_{i}-o_{i}\right)}^{2}}/\left(\max\left\{o_{i}\right\}-\min\left\{o_{i}\right\}\right)$, where m and o are the modelled and observed points, respectively, and N is the number of observed experimental points. It was found that the goodness of fit increases when the value of k increases and the value of *NRMSE* decreases.

**Figure 2:**
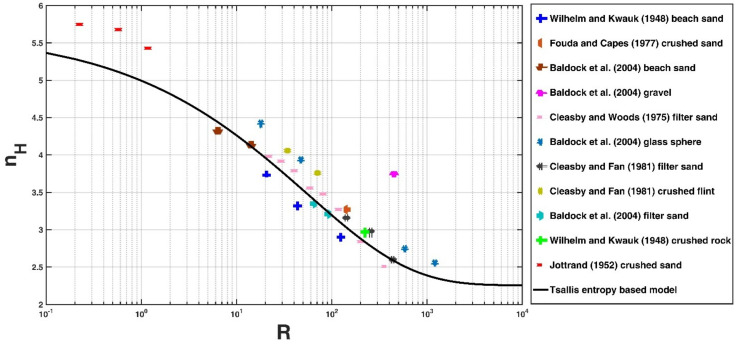
Comparison of the Tsallis entropy-based model (Equation (15)) with the eleven experimental data sets collected.

Figure 2 shows the comparison of the Tsallis entropy-based expression (Equation (15)) with the collected experimental data, and the values of the fitting parameters A and η are taken as 0.08 and 0.45, respectively, after fitting the model with the experimental data. In Equation (15), the values of $\Delta_{p}$ and c are required. For the eleven collected experimental data sets, most of the falling particles have a relative mass density of 2.65, and $\Delta_{p}$= 1.65 was thus adopted in this study. Because the collected experimental data sets are for low as well as high suspension concentrations, an average value of c = 0.2 is taken for the estimation of $n_{H}$ in this study. It can be seen from Figure 2 that the Tsallis entropy-based model shows a good agreement with the experimental data with a high coefficient of determination (k = 0.90) and a low *NRMSE* value (*NRMSE* = 0.10).

## 4. Discussion

### 4.1. Comparison with other Deterministic Models

**Figure 3:**
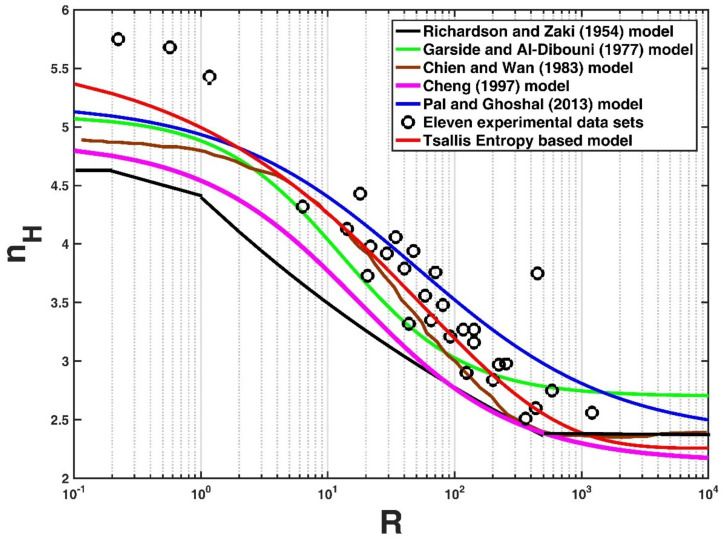
Comparison of five existing deterministic models for $n_{H}$ with R using the eleven experimental data sets collected.

Five existing deterministic models for $n_{H}$ with R in the literature were collected to compare with the developed Tsallis entropy-based model in this study. Table 1 lists the formulations of these models. We compare them with eleven collected experimental data sets regarding the reduction of settling velocity of the falling particle in the literature in Figure 3, as well as the Tsallis entropy-based model developed in this study. The calculated values of k and *NRMSE* for each model are presented in Table 2. It can be seen from this table that the Tsallis entropy-based model has the highest k value and the lowest NRMSE value compared with the other five deterministic models for all of the collected experimental data, even though most of the models had a high coefficient of determination larger than 0.8. Thus, this study shows the potential of the Tsallis entropy together with the principles of maximum entropy to predict the exponent of reduction of the settling velocity of the particle in a particle-fluid mixture.

### 4.2. Comparison with the Shannon Entropy-Based Model

**Figure 4:**
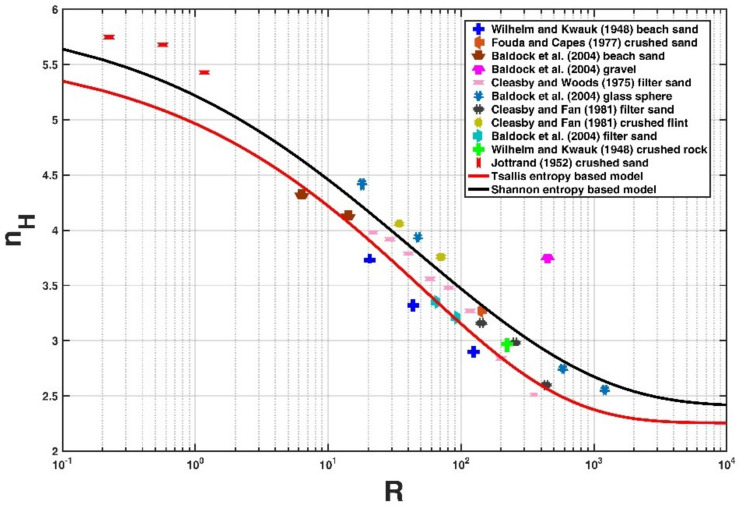
Comparison of the Shannon entropy-based model with the eleven experimental data sets collected, as well as the Tsallis entropy-based model in this study.

We also attempted to compare the Tsallis entropy-based model with the Shannon entropy-based model proposed by Kumbhakar et al. [13] for the collected experimental data in this study, as shown in Figure 4. the calculated value of k and *NRMSE* for the Shannon entropy-based model proposed by Kumbhakar et al. [13] are 0.90 and 0.08 respectively. Comparing the values of k and *NRMSE* corresponding to the Tsallis entropy and the Shannon entropy, respectively, we can conclude that the Tsallis entropy developed in this study is comparable to the Shannon entropy-based model for predicting the hindered settling velocity of a falling particle in a particle-fluid mixture.

### 4.3. Estimation of the Hindered Settling Velocity

Substituting Equation (15) into Equation (1) and fitting the values of A and η, we can obtain the hindered settling velocity of a falling particle in the particle-fluid mixture as follows:

(16) $$\frac{\omega_{m}}{\omega }={\left(1-c\right)}^{-14.29\times {\left\{0.15-0.14\times \exp\left[-0.08{\left(\frac{R}{c\Delta_{p}}\right)}^{0.45}\right]\right\}}^{\frac{2}{3}}+6.29}$$

Regarding the settling velocity of the sediment particle in a clear fluid ω, Song et al. [40] proposed the following expression,

(17) $$\omega =\frac{\nu_{0}}{d_{p}}d_{*}^{3}{\left(38.1+0.93\times d_{*}^{\frac{12}{7}}\right)}^{-\frac{7}{8}},$$

where $d_{*}$ is the non-dimensional particle diameter and can be written as $d_{*}={\left(\frac{\Delta_{p}g}{\nu_{0}^{2}}\right)}^{\frac{1}{3}}d_{p}$, which shows good agreement with the experimental data. Substituting Equation (17) into Equation (16) leads to the expression for the hindered settling velocity of a falling particle in the particle-fluid mixture as follows:

(18) $$\omega_{m}=\frac{\nu_{0}}{d_{p}}d_{*}^{3}{\left(38.1+0.93\times d_{*}^{\frac{12}{7}}\right)}^{-\frac{7}{8}}{\left(1-c\right)}^{-14.29\times {\left\{0.15-0.14\times \exp\left[-0.08\frac{d_{*}^{\frac{27}{20}}{\left(38.1+0.93\times d_{*}^{\frac{12}{7}}\right)}^{-\frac{63}{160}}}{{\left(c\Delta_{p}\right)}^{\frac{9}{20}}}\right]\right\}}^{\frac{2}{3}}+6.29}$$

**Figure 5:**
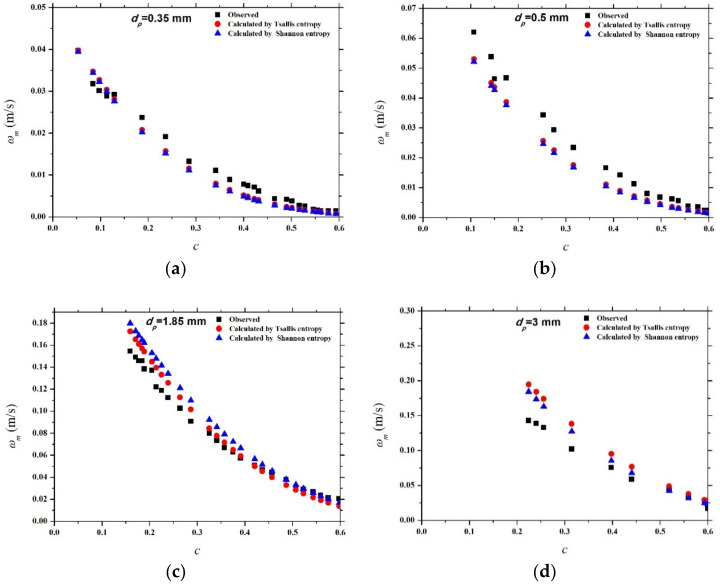

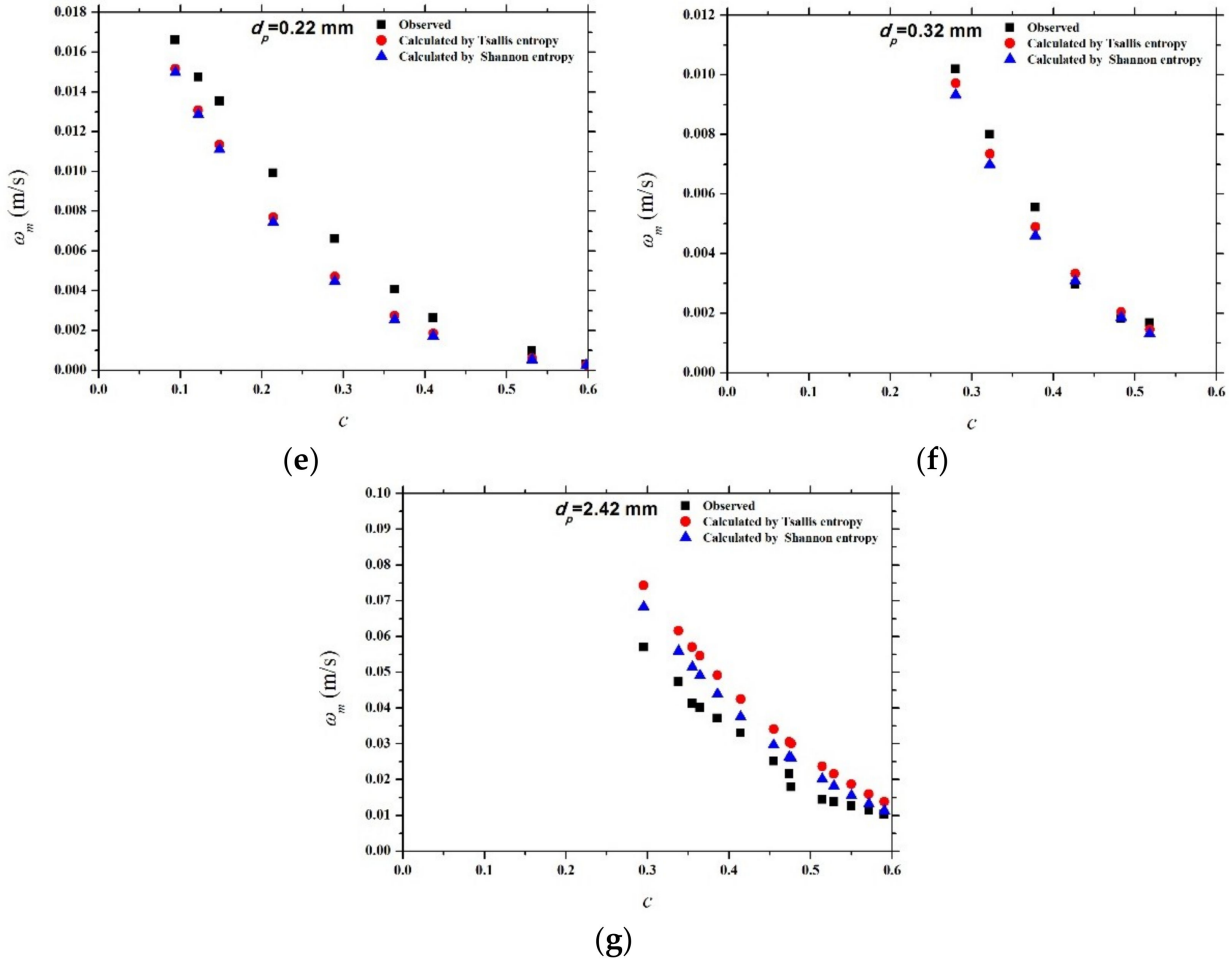
Comparisons between the observed hindered settling velocity values and the calculated settling velocity values based on the Tsallis entropy, as well as the Shannon entropy-based model, for glass particles with particle sizes 0.35 mm (**a**), 0.5 mm (**b**), 1.85 mm (**c**) and 3 mm (**d**), beach sand with particle sizes 0.22 mm (**e**), 0.32 mm and (**f**), gravel with a particle size of 2.42 mm (**g**).

Seven experimental data sets of the settling velocity of Baldock et al. [16] as also presented in the work of Kumbhakar et al. [13], including four data sets of glass particles of various sizes, two sets of beach sand of different sizes, and a set of gravel particles, are adopted in this study to test the validity of Equation (18). Figure 5 shows the comparison results between Equation (18) and each experimental data set, as well as the Shannon entropy-based model proposed by Kumbhakar et al. [13]. Table 3 presents the calculated values of k and *NRMSE* for each case. It could be found that both Equation (18) and the Shannon entropy-based model have a good agreement with the observed values. For five cases of *d_p_* = 0.35 mm, 0.5 mm, 1.85 mm, 0.22 mm and 0.32 mm, Equation (18) has a slightly high prediction accuracy for data sets than the Shannon entropy-based model, whereas the Shannon entropy-based model could provide better results than Equation (18) for two cases of *d_p_* = 3 mm, and 2.42 mm. These show the potential of the proposed Tsallis entropy-based expression, as well as the developed Shannon entropy-based model, to predict the hindered settling velocity of a falling particle in the particle-fluid mixture, as an addition to existing deterministic models.

**Figure 6:**
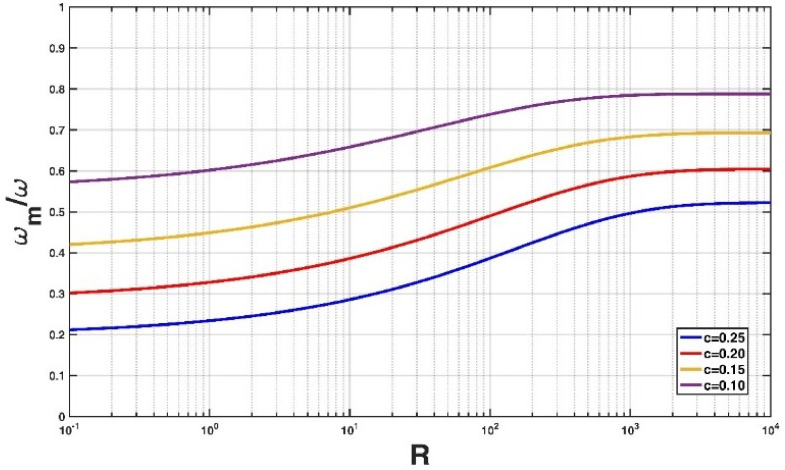
Variation of the non-dimensional hindered settling velocity model (Equation (16)) with R for different values of c.

Figure 6 shows the variation of the non-dimensional hindered settling velocity expression $\omega_{m}/\omega$ (Equation (16)) with R for different values of c. Here, the falling particle is fixed at $\Delta_{p}$ = 1.65. It can be seen that for a given falling particle, the particle settles much more slowly in a high concentration suspension compared to a low concentration suspension. This could be attributed to three reasons: (1) return flow—a falling particle will generate a return flow, for other particles in the vicinity of this falling particle; they are always located within this return flow and thus their settling velocities will be affected; (2) viscosity—the effective viscosity of the suspension will increase with particle concentration; and (3) buoyancy or reduced gravity—the particle settles in the remainder of the suspension with an increased bulk density, and thus the settling velocity decreases, as shown by Winterwerp [9].

**Figure 7:**
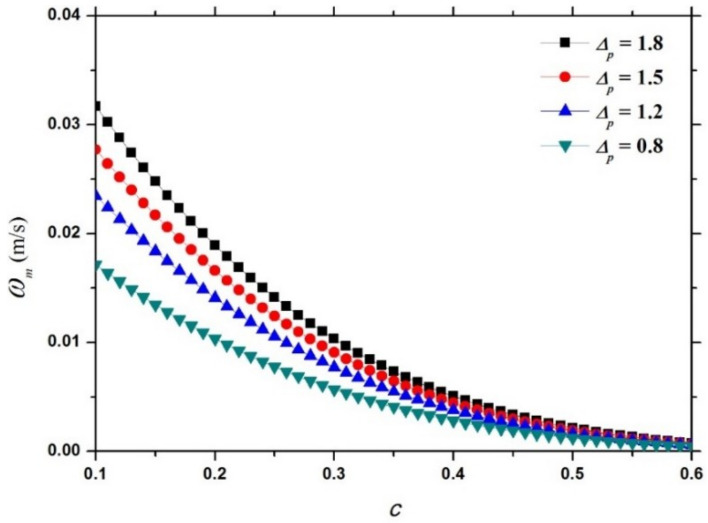
Variation of the dimensional hindered settling velocity model (Equation (18)) with c for different values of$\Delta_{p}$.

**Figure 8:**
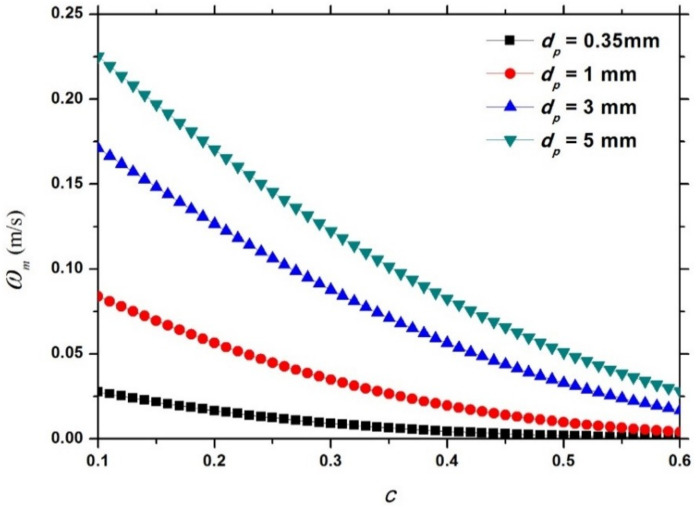
Variation of the dimensional hindered settling velocity model (Equation (18)) with c for different values of $d_{p}$.

Fixing $d_{p}$ at 0.35 mm, Figure 7 shows the variation of the dimensional hindered settling velocity model $\omega_{m}$ (Equation (18)) with c for different values of $\Delta_{p}$. It can be observed that for a falling particle of a given size, a heavy particle settles more rapidly in the particle-fluid mixture than a light particle due to a stronger gravity. Fixing $\Delta_{p}$ at 1.5, Figure 8 shows the variation of the dimensional hindered settling velocity model $\omega_{m}$ (Equation (18)) with c for different values of $d_{p}$. It can be seen that for a falling particle of a given mass density, a large particle settles faster in the mixture than a small particle due to a greater gravity.

Equation (16) provides a new expression for the hindered settling velocity of a falling particle in the particle-fluid mixture based on the Tsallis entropy theory. This expression has a simple mathematical form, contains fewer parameter inputs compared with other deterministic models such as the model of Cheng [12] and the model of Pal and Ghoshal [8] as presented in Table 1. However, it should be noted that some physical properties present in some existing deterministic models are not incorporated into the developed Tsallis entropy-based expression. For example, the effect of the suspension concentration on the kinematic viscosity of the sediment-fluid mixture has been incorporated into the model of Pal and Ghoshal [8], which introduced the maximum volumetric concentration of the suspended particle $c_{\max}$. However, the entropy-based expression does not contain this parameter.

## 5. Conclusions

This study attempts to derive an expression for the exponent of reduction of the settling velocity of a falling particle in a particle-fluid system based on the Tsallis entropy theory together with the principle of maximum entropy. The function of the exponent of the reduction of the settling velocity is derived by assuming that this exponent is a random variable, maximizing the Tsallis entropy function subject to two constraint equations and using the hypothesis regarding the cumulative distribution function of this exponent. The derived expression is a function of the volumetric concentration of the suspended particle, the relative mass density of the particle, and the particle’s Reynolds number.

The Tsallis entropy-based expression is tested against eleven collected experimental data sets. Error analysis indicates that the model has a good agreement with the experimental data. The model is also compared with five existing deterministic models for the collected experimental data, and it shows better prediction accuracy compared to other deterministic models. Furthermore, the derived model is also compared with the existing Shannon entropy-based model for the collected experimental data, and the Tsallis entropy is comparable to the Shannon entropy-based model for predicting the hindered settling velocity of a falling particle in a particle-fluid mixture. Finally, an empirical expression for the hindered settling velocity is proposed, which shows high prediction accuracy for the experimental data regarding the hindered settling velocity. This study indicates the potential of using Tsallis entropy together with the principle of maximum entropy to predict the hindered settling velocity of a falling particle in a particle-fluid mixture.

## Figures and Tables

**Table 1:** Formulations of five existing deterministic models for $n_{H}$

Model Name	Formulation
Richardson and Zaki [11] model	$n_{H}$= 4.65, for R < 0.2
	$n_{H}$= 4.4×$R^{-0.03}$, for 0.2 < R < 1
	$n_{H}$= 4.4×$R^{-0.1}$, for 1 < R < 500
	$n_{H}$= 2.4, for R > 500
Garside and Al-Dibouni [14] model	$n_{H}$= $\frac{5.1+0.27\times R^{0.9}}{1+0.1\times R^{0.9}}$
Chien and Wan [15] model	$n_{H}$= 4.91 at low R
	$n_{H}$is determined by graphical curve at moderate R
	$n_{H}$= 2.25 at highR
Cheng [12] model	$n_{H}$=$\frac{\begin{array}{l} \ln(\frac{2-2c}{2-3c})+1.5\times \ln\left\{\frac{-5+\sqrt{25+{\left[\frac{\left(1-c\right){\left(2-3\times c\right)}^{2}}{4+4\Delta_{p}c}\right]}^{\frac{2}{3}}\left(R^{\frac{4}{3}}+10\times R^{\frac{2}{3}}\right)}}{-5+\sqrt{25+R^{\frac{4}{3}}+10\times R^{\frac{2}{3}}}}\right\} \end{array}}{\ln(1-c)}$
Pal and Ghoshal [8] model	$n_{H}$=$\frac{\frac{4}{3}\times \ln(1-c)-\ln(1-\frac{c}{c_{\max}})+3\times \ln f-\frac{7}{8}\times \ln\left(\frac{38.1+5.74\times f^{\frac{12}{7}}R^{\frac{4}{7}}}{38.1+5.74\times R^{\frac{4}{7}}}\right)}{\ln(1-c)}$, where $f={\left[\frac{\left(1+\Delta_{p}\right){\left(1-c\right)}^{\Delta_{p}}-1}{\Delta_{p}}{\left(1-\frac{c}{c_{\max}}\right)}^{2}{\left(1-c\right)}^{-1}\right]}^{\frac{1}{3}}$, $c_{\max}$ is the maximum volumetric concentration of suspended particle

**Table 2:** Error analysis between five existing deterministic models and the eleven experimental data sets collected.

Model Name	k	*NRMSE*
Richardson and Zaki [11] model	0.88	0.22
Garside and Al-Dibouni [14] model	0.87	0.15
Chien and Wan [15] model	0.81	0.15
Cheng [12] model	0.88	0.21
Pal and Ghoshal [8] model	0.86	0.14
The Tsallis entropy-based model	0.90	0.10

**Table 3:** Error analysis between the observed settling velocity values and the calculated settling velocity values by the Tsallis entropy and the Shannon entropy.

Experimental Data Sets	Model Name	k	*NRMSE* (×10^−2^)
*d_p_* = 0.35mm	Tsallis entropy-based model	9.840	5.163
	Shannon entropy-based model	9.835	5.683
*d_p_* = 0. 5mm	Tsallis entropy-based model	9.890	8.484
	Shannon entropy-based model	9.851	9.498
*d_p_* = 1.85mm	Tsallis entropy-based model	9.977	7.193
	Shannon entropy-based model	9.971	10.770
*d_p_* = 3mm	Tsallis entropy-based model	9.953	23.350
	Shannon entropy-based model	9.944	17.200
*d_p_* = 0.22mm	Tsallis entropy-based model	9.913	9.287
	Shannon entropy-based model	9.892	10.460
*d_p_* = 0.32mm	Tsallis entropy-based model	9.966	5.470
	Shannon entropy-based model	9.954	8.093
*d_p_* = 2.42mm	Tsallis entropy-based model	9.908	20.070
	Shannon entropy-based model	9.914	13.960
